# Radiological and clinical features of large consolidative‐type pulmonary invasive mucinous adenocarcinoma

**DOI:** 10.1111/crj.13743

**Published:** 2024-03-26

**Authors:** Jiaqi Chen, Linlin Qi, Jianwei Wang, Liyan Xue, Qi Xue, Jia Jia, Guochao Zhang, Jianing Liu, Fenglan Li, Shulei Cui

**Affiliations:** ^1^ Department of Diagnostic Radiology National Cancer Center/National Clinical Research Center for Cancer/Cancer Hospital, Chinese Academy of Medical Sciences and Peking Union Medical College Beijing China; ^2^ Department of Diagnostic Pathology National Cancer Center/National Clinical Research Center for Cancer/Cancer Hospital, Chinese Academy of Medical Sciences and Peking Union Medical College Beijing China; ^3^ Department of Thoracic Surgery National Cancer Center/National Clinical Research Center for Cancer/Cancer Hospital, Chinese Academy of Medical Sciences and Peking Union Medical College Beijing China

**Keywords:** computed tomography, mucinous adenocarcinoma, pathology, prognosis, radiology

## Abstract

**Background:**

This study aimed to investigate the radiological, pathological, and prognostic characteristics of large consolidative‐type pulmonary invasive mucinous adenocarcinomas (IMA).

**Methods:**

We retrospectively reviewed 738 patients who confirmed IMA between January 2010 and August 2022, and two radiologists reviewed imaging data to determine subtypes. We included 41 patients with pathologically large consolidative‐type IMA. We analyzed their radiological, pathological, and prognostic characteristics. The recurrence‐free survival (RFS) and overall survival (OS) were determined using the Kaplan–Meier method.

**Results:**

Most lesions were located in the lower lobe, with 46.3% patients showing multiple lesions. Halo, angiogram, vacuole, air bronchogram, and dead branch sign were observed in 97.6%, 73.2%, 63.4%, 61.0%, and 61.0% of the cases, respectively. Unevenly low enhancement was observed in 88.89% of patients. T3 and T4 pathological stages were observed in 50.0% and 30.6% of patients, respectively. Lymph node metastasis was observed in 16.7% patients, with no distant metastasis. Spread‐through air spaces and intrapulmonary dissemination were observed in 27.8% and 19.4% patients, respectively. Moreover, Kirsten rat sarcoma viral oncogene mutations were found in 68.6% of cases, and no epidermal growth factor receptor mutations were seen. Among all mutation sites, G12V mutation is the most common, accounting for 40%. The average RFS and OS were 19.4 and 66.4 months, respectively, with 3‐year RFS and OS rates of 30.0% and 75.0%, respectively. Pleural invasion and lymph node metastasis were independent risk factors for diagnosis.

**Conclusion:**

Halo, vacuole, angiogram, and dead branch signs were frequently observed in consolidative‐type IMA. Kirsten rat sarcoma viral oncogene mutations are common in consolidative‐type IMA, especially site G12V, whereas epidermal growth factor receptor mutations were rare; therefore, gene immunotherapy was more difficult. Most patients were in stage T3–T4; however, lymph node metastasis was rare.

## Graphical abstract

Invasive mucinous adenocarcinoma (IMA) accounts for approximately 2%–10%, and large consolidative‐type IMA has a low clinical incidence, which has diverse radiological manifestations, with halo, vacuole, angiogram, and dead branch signs being frequently observed. The T stage of large consolidative‐type IMA is relatively late, and lymph node metastasis is rare. KRAS mutations were common in consolidative‐type IMA, whereas EGFR mutations were rare. Consolidative‐type IMA has a poor prognosis.

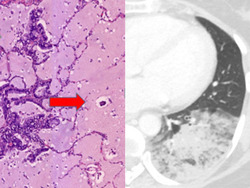

## 1 INTRODUCTION

Worldwide, lung cancer has the highest mortality rate and the second‐highest incidence rate after breast cancer.^1^ Adenocarcinoma is the most common pathological pattern. Invasive adenocarcinomas can be classified as mucinous adenocarcinoma (IMA) and non‐mucinous adenocarcinoma.^2^ IMA was once known as mucinous bronchioloalveolar carcinoma. In 2011, the International Association for Lung Cancer Research/American Thoracic Society/European Respiratory Society International Multidisciplinary Classification of Lung Adenocarcinoma completely discontinued the use of “bronchioloalveolar carcinoma” and proposed using “IMA.”^3^ The 5th edition of the World Health Organization classification of tumors continued this use.^4^ IMA accounts for approximately 2%–10% of lung adenocarcinomas.^5^ Pathologically, IMA tumor cells show goblet and/or columnar morphology with abundant intracytoplasmic mucin and small, basally oriented nuclei. Nuclear atypia is usually inconspicuous or absent. The surrounding alveolar spaces are often mucin‐filled.^4^ Based on the percentage of mucinous type cells, IMA can be classified as a pure mucinous type (invasive mucinous type > 90%) or mixed mucinous/non‐mucinous type (non‐mucinous invasive component ≥ 10%).^6^ Radiologically, IMA has diverse imaging manifestations, including low density, vacuoles, halos, air bronchograms, and spiculation.^7^, ^8^ Using computed tomography (CT) data, IMA can be divided into the nodular/mass and consolidative/pneumonic types, with the former being common and the latter being rare. The consolidative/pneumonic type is often misdiagnosed as an inflammatory disease, leading to delayed treatment and worsening prognosis. However, few studies have focused on the consolidative/pneumonic type of IMA. Accordingly, this study aimed to investigate the radiological, pathological, and prognostic characteristics of large consolidative‐type pulmonary IMA. This could improve diagnosis, treatment, and follow‐up for patients with consolidative‐type IMA.

## 2 MATERIALS AND METHODS

This retrospective study was approved by the ethics committee of our institution, which waived the requirement for informed consent.

### 2.1 Patients

**FIGURE 1:**
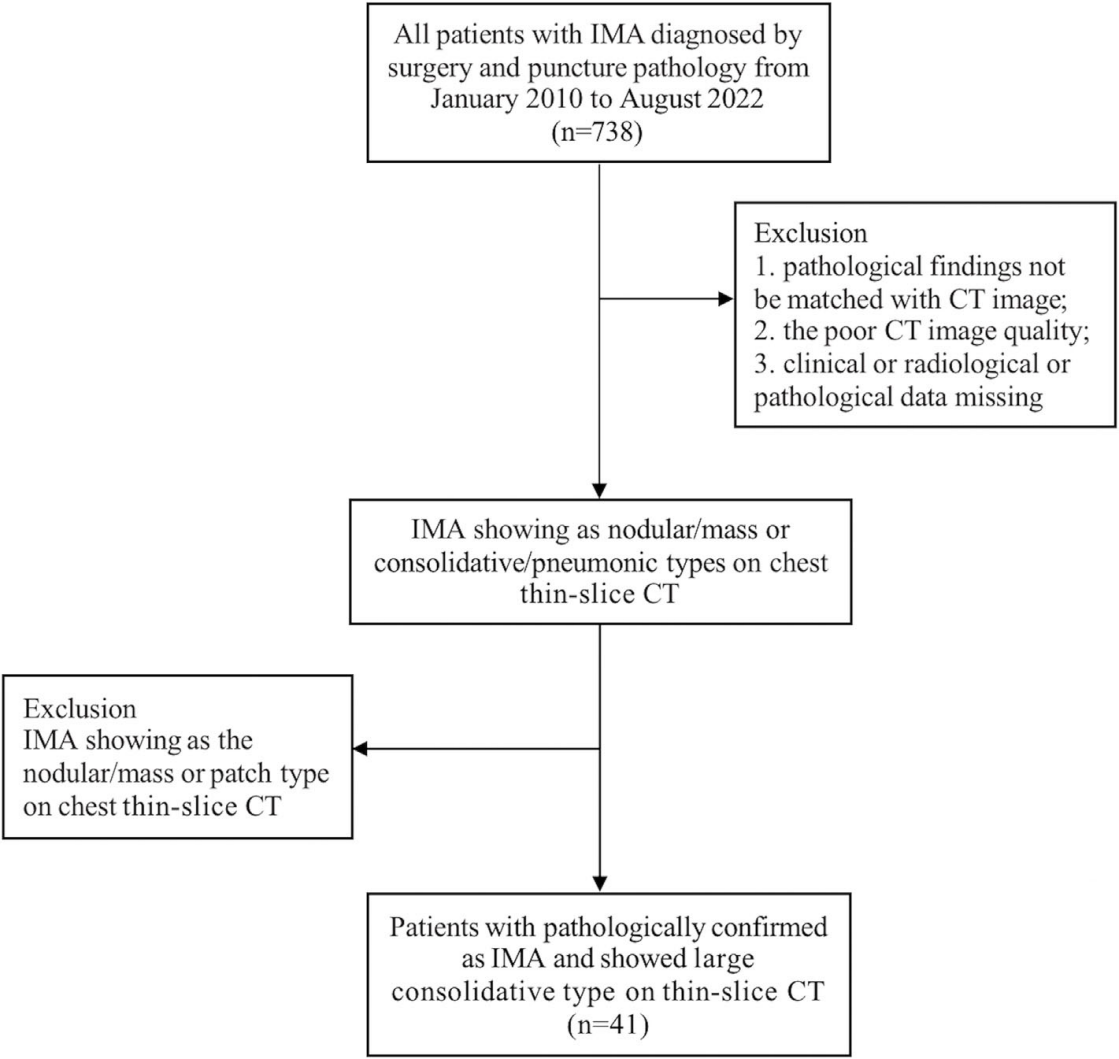
Flowchart of the study population. Numbers in parentheses are the numbers of patients.

We retrospectively reviewed patients with pathologically confirmed IMA between January 2010 and August 2022. The inclusion criteria were as follows: (1) IMA diagnosed through surgery or puncture biopsy and (2) large consolidative‐type features on chest thin‐slice CT within 1 month prior to surgery. The exclusion criteria were as follows: (1) chest thin‐slice CT showing the IMA as a nodular/mass or patch type; (2) pathological findings not accurately matching the chest CT findings; (3) poor CT image quality; and (4) missing clinical, radiological, or pathological data (Figure 1). We collected 738 cases of IMA, and after reviewing imaging data, finally, 41 cases of large consolidative‐style IMA pathologically and radiologically were included in the study. In this study, patients received neoadjuvant chemotherapy or postoperative chemotherapy, using cisplatin/carboplatin + pemetrexed/paclitaxel as the medication. Only one patient with bone metastasis received four courses of radiation therapy without undergoing chemotherapy.

### 2.2 CT scanning protocol

A 64‐row spiral CT scanner (LightSpeed VCT, Discovery CT750 HD, or Optima CT66; GE HealthCare, Chicago, IL, USA) was used. The CT parameters were as follows: tube voltage, 120 kVp; auto mA settings, tube current, approximately 200–350 mA; noise index, 13; pitch, 0.992 or 0.984; rotation time, 0.5 s; thickness, 5 mm; lung window width of 1600 Hounsfield units (HU) and level of −600 HU; and mediastinal window width of 360 HU and level of 60 HU. The reconstruction thicknesses were 1.25 or 1.0 mm; moreover, the intervals were 0.8 mm using a standard reconstruction algorithm. For enhanced CT scans, iopromide (iodine concentration, 300 mg/mL) was injected at a dose of 80–90 mL and a flow rate of 2.5 mL/s and used as the contrast agent. Subsequently, images were obtained 35 s after contrast agent injection.

### 2.3 Analyses of clinical and radiological characteristics

**FIGURE 2:**
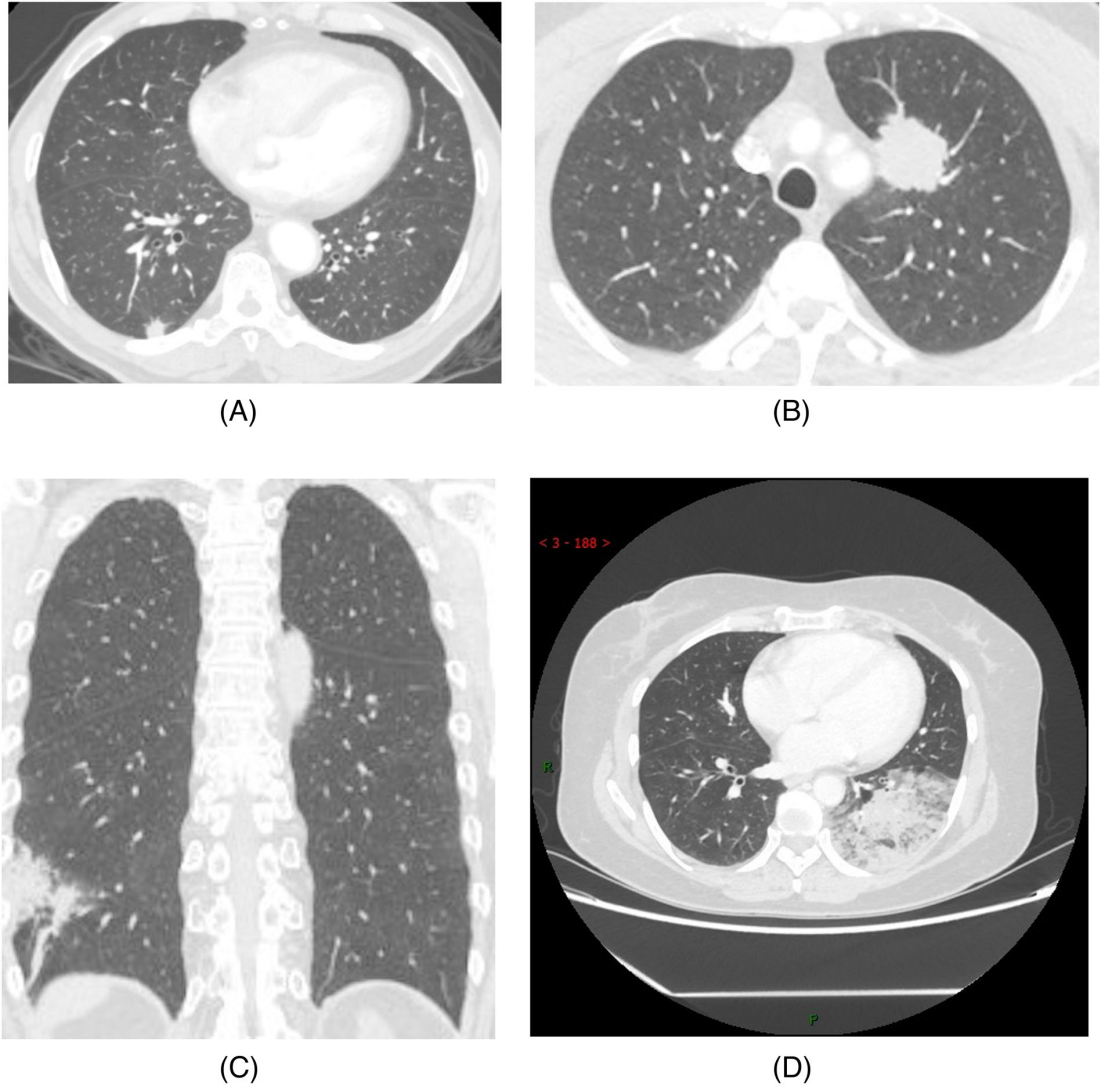
The CT imaging classification of invasive mucous adenocarcinoma (IMA). (A) CT imaging of nodular‐type IMA (*d* < 3 cm), (B) mass‐type IMA (*d* ≥ 3 cm), (C) patch‐type IMA, and (D) large consolidative‐type IMA.

**FIGURE 3:**
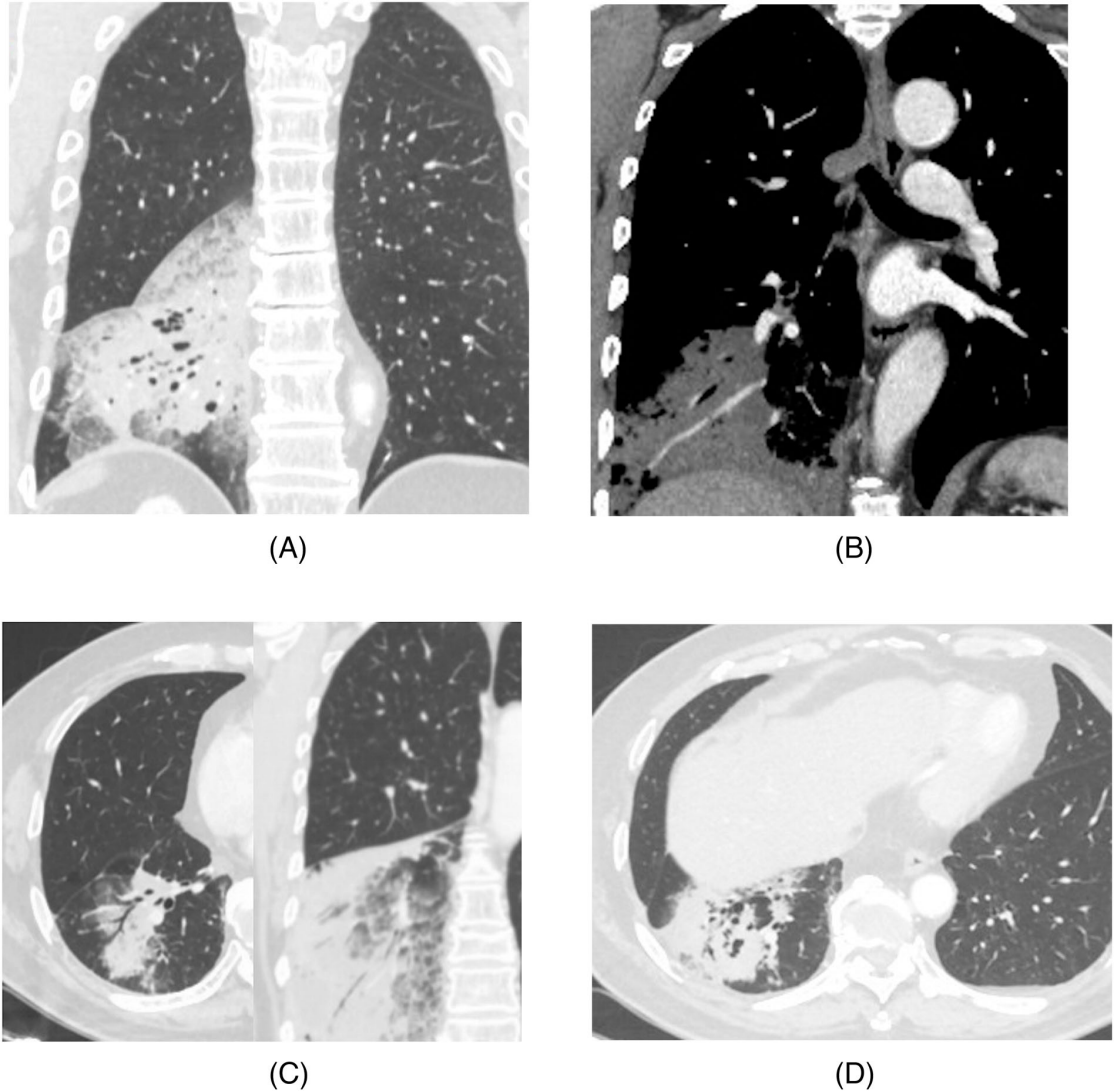
The CT features of large consolidative‐type invasive mucinous adenocarcinoma (IMA). (A) Halo sign refers to the ground glass shadow around the lesion, which results from the infiltration of a large amount of mucus and macrophages around the lesion area. (B) Angiogram sign refers to obvious enhancement of vascular shadow passes through the low‐density consolidation image on the enhanced CT. (C) Air bronchus and dead branch sign mean that the bronchus shadow can be seen in the consolidation area. The smooth bronchus is called the “air bronchus,” and the twisted and invaded bronchus is called “dead branch sign.” (D) Vacuole sign refers to the gas‐containing part of the lung with a diameter usually <5 mm. A cavity is produced by the expulsion or drainage of a necrotic part of the lesion via the bronchial tree with a diameter usually >5 mm.

**FIGURE 4:**
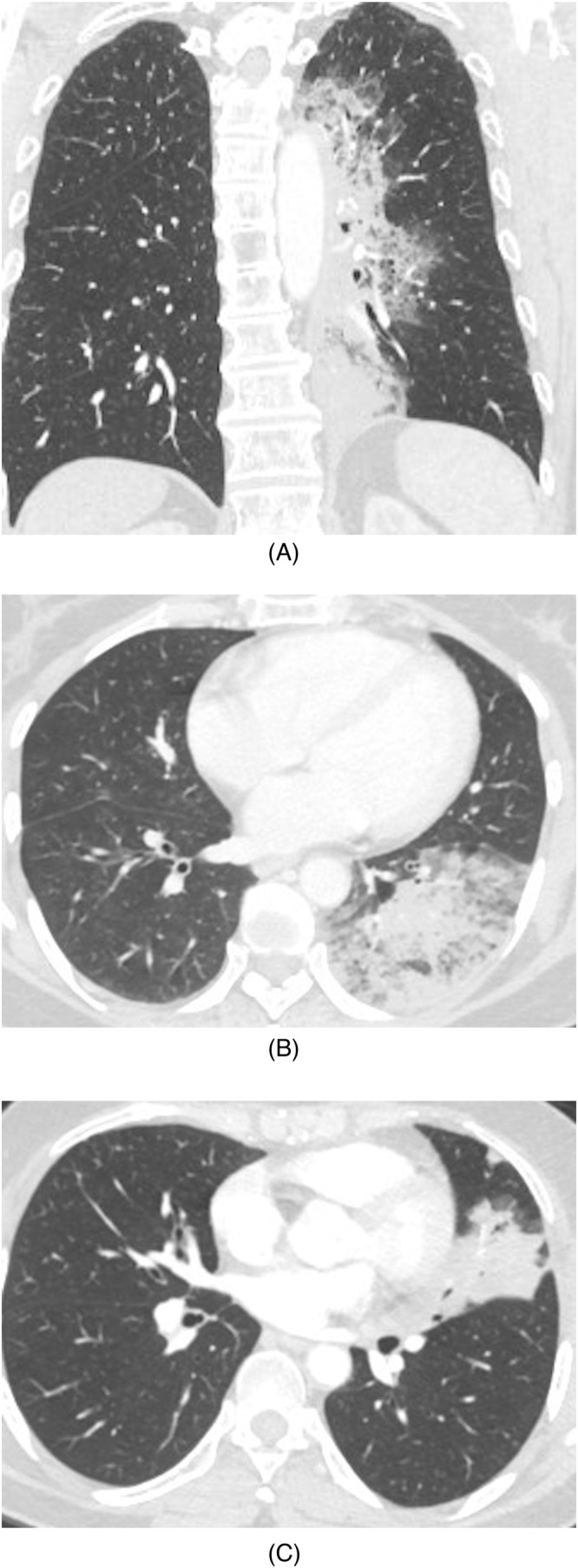
The large consolidative‐type invasive mucinous adenocarcinoma (IMA) was defined as follows: (A) the lesion occupied at least one lung lobe; (B) the main lesion volume accounted for 1/5 or more of the lung lobe volume; or (C) the main lesion crossed the inner, middle, and outer zones of the lung lobe.

The clinical and radiological characteristics of the included patients were reviewed. The clinical characteristics included age, sex, symptoms, smoking history, malignancy history, diagnosis method, and operation duration. CT features included the lesion type (nodular/mass/patch/large consolidative type) (Figure 2), location, number (single or multiple), volume proportion of lung lobes occupied, and morphological features (including halo sign, dead branch sign, angiogram sign, vacuole sign, and cavity sign; Figure 3). The definition criteria for large consolidative‐type IMA were (1) the lesion occupied at least one lung lobe; (2) the main lesion volume accounted for 1/5th or more of the lung lobe volume; or (3) the main lesion crossed the inner, middle, and outer zones of the lung lobe (Figure 4). The aforementioned CT characteristics were analyzed on multiplanar reconstruction images by two radiologists with 2 and 7 years of experience in chest CT. Disagreements were resolved through consultation with a senior radiologist with 25 years of experience in chest CT.

### 2.4 Pathological analysis

A senior pathologist with 20 years of experience reviewed histopathology slides of samples from all eligible patients based on the 2021 World Health Organization classification of lung adenocarcinomas and the 8th edition TNM staging of lung cancer. In this study, professional pathologists used elastic fiber staining to determine whether there was pleural invasion. Continuous pleural elastic fibers could be seen by staining with pleural elastic fibers (without pleural invasion). When pleural invasion occurs, it manifests as discontinuous elastic fibers. Moreover, molecular analyses of mutations of epidermal growth factor receptor (EGFR) and Kirsten rat sarcoma viral oncogene (KRAS) were performed using a polymerase chain reaction‐based amplification refractory mutation system with the Human EGFR Gene Mutations Detection Kit (Beijing ACCB Biotech Ltd., Beijing, China).

### 2.5 Follow‐up

Postoperative follow‐up data were obtained from medical records or telephone interviews. The follow‐up endpoint was May 30, 2022. Recurrence‐free survival (RFS) was defined as the period from the operation date to the date of recurrence or death from any cause or the follow‐up endpoint. Recurrence referred to recurrence adjacent to the surgical area, pulmonary recurrence, mediastinal recurrence, or distant recurrence. Overall survival (OS) was defined from the operation date to the date of death from any cause. We calculated the 3‐year RFS and OS rates for patients who were followed up for ≥3 years.

### 2.6 Statistical analyses

All statistical analyses were performed using SPSS software (v23.0; IBM Corp., Armonk, NY, USA). Normally and non‐normally distributed continuous variables are expressed as mean ± standard deviation and median (range), respectively. Categorical data are presented as numbers (percentages). The Kaplan–Meier method was used to calculate the cumulative survival rate and plot survival curves. Univariate analyses were used to identify prognostic factors for IMA using Kaplan–Meier analysis with the log‐rank test. To determine independent predictors of IMA prognosis, we performed multivariate Cox proportional hazards regression with forward stepwise selection, with adjustment for variables with *P*‐values of <0.20 in the univariate analyses. Statistical significance was set at *P* < 0.05.

## 3 RESULTS

### 3.1 Clinical features

Among the 41 included patients, there were 22 women and 19 men (mean age: 61.00 ± 9.31 years [range: 37–78 years]). Only one patient had a history of malignancy (lung adenocarcinoma); further, 13 patients had a smoking history. There were clinical symptoms in 24 (58.54%) patients, including cough (*n* = 24), sputum (*n* = 14), and hemoptysis (*n* = 7). Finally, diagnosis was confirmed in 36 and five patients by surgical pathology and CT‐guided aspiration biopsy, respectively (Table 1).

**TABLE 1:** Clinical and radiological characteristics of large consolidative‐type IMA.

Clinical features	
Age (years)	61.00 ± 9.31
Gender	
Male	19 (46.34%)
Female	22 (53.66%)
Smoking history	13 (31.71%)
Symptom	
Coughing	24 (58.54%)
Sputum	14
Hemoptysis	7
History of Malignancy	1
Diagnosis method	
Surgery	36
Puncture biopsy	5
Radiological features	
Location	
Right	
Upper	3
Middle	4
Lower	14
Left	
Upper	4
Lower	20
Both lungs	1
Number of lesions	
Single lesion	22 (53.66%)
Multiple lesions	19 (46.34%)
Proportion of lung lobe occupied	
Unilateral lung	38
Single lobe	35
1/5	1
1/4	4
1/3	9
1/2	11
2/3	6
1	4
Multiple lobes	3
Bilateral lung	3
Average diameter (cm)	8.90 ± 2.59
Morphological characteristics	
Pleural retraction	40 (97.56%)
Halo sign	40 (97.56%)
Angiogram sign	30 (73.17%)
Vacuole sign	26 (63.41%)
Dead branch sign	25 (60.98%)
Air bronchogram sign	25 (60.98%)
Cavities	9 (21.95%)
Necrosis	5 (12.20%)
Calcification	2 (4.88%)
Pleural effusion	1 (2.44%)
Enhancement patterns	
Low enhancement	3 (8.33%)
Uneven low enhancement	32 (88.89%)
Significant enhancement	1 (2.78%)

*Note*: Values are expressed as number (%), mean ± standard deviation, or median (range).

### 3.2 Radiological features

Table 1 presents the radiological characteristics of the patients with large consolidative‐type IMA. The lesions were mostly located in the bilateral lower lung lobes (35/41, 85.37%). CT imaging revealed single and multiple lesions in 22 (53.66%) and 19 (46.34%) patients, respectively. Additionally, 38 patients showed involvement of unilateral lung fields (including 35 and three patients with involvement of one and two lung lobes, respectively), and three patients showed involvement of bilateral lung fields. Among the 35 cases involving a single lung lobe, 1, 4, 9, 11, 6, and 4 cases showed involvement of 1/5th, 1/4th, 1/3rd, 1/2, 2/3, and a full lung lobe, respectively. Among the three cases involving bilateral lungs, two and one involved two and five lobes, respectively. The mean diameter of the main lesions was 8.90 ± 2.59 cm. Regarding the morphological features, halo, angiogram, vacuole, air bronchogram, and dead branch sign were observed in 40 (97.56%), 30 (73.17%), 26 (63.41%), 25 (60.98%), and 25 cases (60.98%), respectively; moreover, cavities and necrosis were observed in nine (21.95%) and five (12.20%) cases. Only two (4.88%) and one (2.44%) case had calcification and pleural effusion, respectively.

### 3.3 Pathological features and genetic testing

**FIGURE 5:**
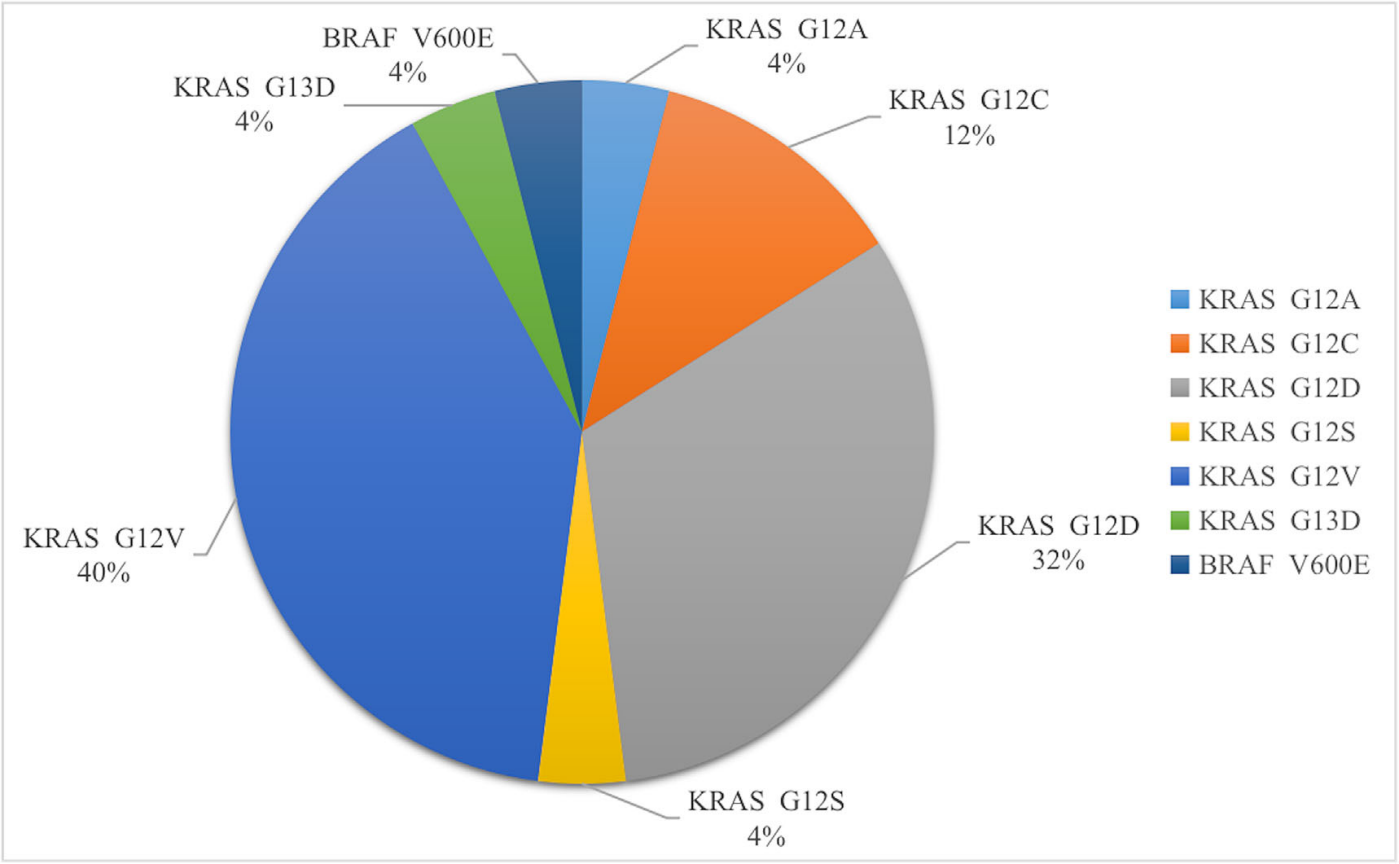
Pie chart of the gene mutation points in this study. The percentage on the chart is the percentage of patients with mutations at this site.

IMAs contained basal, ciliated columnar epithelial, and mucus cells. Surgical resection was performed on 36 patients; among them, 7, 18, and 11 cases were in the T2, T3, and T4 stages, respectively. Further, 16.67% (6/36) of these patients presented lymph node metastasis (N1 stage, *n* = 4; N2 stage, *n* = 2), whereas 83.33% (30/36) had no lymph node metastasis. There were no cases of distant metastases. Pleural invasion, spread‐through air spaces (STAS), and intrapulmonary dissemination were observed in 33.33% (5/15, 21 not tested), 27.78% (10/36), and 19.44% (7/36) of the patients, respectively. In this study, 21 cases did not undergo elastic fiber staining. Additionally, 35 patients underwent genetic testing; among them, 24 (68.57%), 1 (2.86%), and 0 (0%) showed KRAS, vrafmurine sarcoma viral oncegene homolog B (BRAF), and EGFR mutations, respectively (Table 2). Further analysis of the gene mutation sites showed six KRAS mutation sites; among them, 1 (4%), 3 (12%), 8 (32%), 1 (4%), 10 (40%), and 1 (4%) showed G12A, G12C, G12D, G12S, G12V, and G13D mutation sites, respectively (Figure 5).

**TABLE 2:** Pathological characteristics and genetic testing of large consolidative‐type IMA.

Pathological features and genetic testing	
Intrapulmonary dissemination	
Yes	7 (19.44%)
No	29 (80.56%)
Pleural invasion	
Yes	5 (33.33%)
No	10 (66.67%)
Not tested	21
STAS	10 (27.78%)
Pathological maximum diameter (cm)	8.81 ± 2.40
Pathological staging	
T	
2	7 (19.44%)
3	18 (50.00%)
4	11 (30.56%)
N	
0	30 (83.33%)
1	4 (11.11%)
2	2 (5.56%)
M	
0	36 (100%)
Genetic testing	
EGFR mutation	0 (0%)
KRAS mutation	24 (68.57%)

*Note*: Values are expressed as number (%), mean ± standard deviation, or median (range).

Abbreviations: BRAF, vrafmurine sarcoma viral oncegene homolog B; EGFR, epidermal growth factor receptor; KRAS, Kirsten rat sarcoma viral oncogene; STAS, spread through air spaces.

### 3.4 Prognostic analysis

Among the 36 patients who underwent surgical resection, five were lost to follow‐up. The median follow‐up duration of the remaining 31 patients was 35.5 months (6.5–95.6 months). During follow‐up, 19 cases showed progression, including 15, 1, 1, 1, and 1 case of intrapulmonary, liver, brain, bone, and peritoneal metastasis, respectively. Moreover, eight patients died during follow‐up. The median RFS and mean OS of the included patients were 19.37 months (1.13, 86.80) and 66.42 ± 5.75 months, respectively. Twenty patients were followed up for ≥3 years. The 3‐year RFS and OS rates were 30.0% and 75.0%, respectively (Table 3).

**TABLE 3:** Prognosis of large consolidative‐type IMA.

Prognosis	
Progress	
No	12 (34.48%)
Yes	19 (65.52%)
Lung	15
Brain	1
Liver	1
Bone	1
Peritoneum	1
Death	
No	23 (74.19%)
Yes	8 (25.81%)
RFS (months)	19.37 (1.13,86.80)
3 years RFS (%)	30.00%
OS (months)	66.42 ± 5.75
3 years OS (%)	75.00%

*Note*: Values are expressed as number (%), mean ± standard deviation, or median (range).

Abbreviations: OS, overall survival; RFS, relapse‐free survival.

Follow‐up patients will be divided into low‐risk and high‐risk groups based on their progression, with the low‐risk group indicating no progression (*n* = 12) and the high‐risk group indicating progression (*n* = 19). We performed univariate and multivariate analyses on patients followed up (*n* = 31) to identify prognostic factors for IMA (Table 4). Multivariate analysis revealed that pleural invasion (*p* = 0.002) and lymph node metastasis (*p* = 0.019) were independent risk factors for diagnosis of large consolidated‐type IMA (Table 4).

**TABLE 4:** Univariate and multivariate analyses of the prognosis of large consolidative‐type IMA.

	Low‐risk group (no progress, *N* = 12)	High‐risk group (Progress, *N* = 19)	*P*‐value	Univariate analysis	Multivariate analysis		
				*P‐*value	*P‐*value	Exp(B)	95% CI
Gender			0.756	0.915			
Female	7	10					
Male	5	9					
Age	62.08 ± 10.79	59.11 ± 7.65	0.236	***0.003***			
Smoking history	2	7	0.418	0.434			
Symptoms	5	13	0.141	0.328			
Percentage of lung lobes	1/3 (1/4,2/3)	1/2 (1/5,2)	0.12	0.219			
Air bronchogram sign	6	12	0.47	0.500			
Vacuole sign	9	13	0.694	0.777			
Cavities	2	7	0.418	0.351			
Necrotic	2	2	0.619	0.586			
Pleural retraction	12	18	0.419	0.955			
Halo sign	12	19					
Angiogram sign	8	15	0.447	0.749			
Dead branch sign	6	12	0.470	0.701			
Multifocal	7	7	0.242	0.891			
Calcification	1	1	0.735	0.260			
Average diameter	8.033 ± 1.281	9.097 ± 2.538	***0.022***	***0.000***			
Pathological max diameter	8.200 ± 1.842	9. 116 ± 2.845	***0.046***	***0.034***			
Intrapulmonary dissemination	2	3	0.948	0.647			
Pleural invasion	0	4	0.221	***0.004***	***0.002***	***0.108***	***0.027, 0.434***
T Staging			0.112	***0.127***			
T2	1	6					
T3	9	7					
T4	2	6					
Lymph node metastasis	1	4	0.624	***0.068***	***0.019***	***0.224***	***0.064, 0.780***
STAS	2	6	0.433	***0.149***			
KRAS mutation	8	13	0.831	0.389			

*Note*: Values are expressed as number (%), mean ± standard deviation, or median (range).

Abbreviations: KRAS, Kirsten rat sarcoma viral oncogene; STAS, spread through air spaces.

## 4 DISCUSSION

We investigated the clinical, radiological, and pathological characteristics of large consolidative‐type IMA. Large consolidative‐type IMA has a low clinical incidence. We found that IMA was most common in the elderly population. Further, we observed diverse radiological manifestations, including halo, vacuole, angiographic, and dead branch sign as well as multifocality. KRAS and EGFR mutations were common and rare, respectively, in consolidative‐type IMA. Upon initial diagnosis of IMA, the pathological stage was usually T3–T4; contrastingly, lymph node metastasis and distant metastasis were rare. Taken together, consolidative‐type IMA has a poor prognosis.

In our study, IMA was mostly located in the lower lobe (35/41, 85.37%), which could be attributed to gravity. IMA has diverse radiological manifestations, with halo, angiographic, and dead branch sign frequently observed and cavities rarely occurring, consistent with previous studies.^8^, ^9^, ^10^, ^11^, ^12^ However, recent studies have demonstrated that these CT features lack specificity and can be observed in lymphoma or pneumonia, which impedes differentiation of IMA. IMA and pneumonia are differentiated mainly based on clinical symptoms and detailed CT features. Compared with IMA, pneumonia involves a shorter disease course and often presents fever symptoms. Further, on CT imaging, lobar pneumonia shows consolidative shadows without bronchial involvement, in which an air bronchogram sign can be observed; contrastingly, IMA shows a dead branch sign. Compared with IMA, pneumonia often involves a significantly larger consolidation area,^13^ and lung lymphoma mainly grows along the vascular and bronchial bundles, with frequent dilation of the bronchi and rare occurrences of a bronchial air sign.^14^

Pathologically, the IMA originates from the goblet and columnar epithelial cells of the alveolar wall.^15^ These cells undergo cancerization and produce a large mucus amount to form a mucus lake, leading to alveolar cavity expansion.^16^ Tumor cells that survive with mucus pass through the alveolar wall and spread to other lung regions along the airway, which may lead to the formation of a large consolidative IMA.^17^ No clear studies have shown that nodular‐type IMA may be the early stage of large consolidative‐type IM, and our own has not observed cases of nodules developing into large consolidation. This suggests that large consolidative‐type IMA may be an independent IMA subtype.

We found that 19.44% of cases had intrapulmonary dissemination. The dissemination mechanisms of lung cancer include hematogenous dissemination, lymphatic metastasis, direct invasion, and STAS.^16^ STAS has a relatively high incidence among IMA cases, which accounted for 27.78% of our cases. In our study, 46.34% of the IMA cases showed multiple lesions, which could be attributed to the spread of tumor cells to other lung tissues along the small airways.^12^, ^17^ Other studies have demonstrated that mucin (MUC5AC) is crucially involved in the formation of multifocal IMA,^18^ which requires further research.

We found that KRAS mutations were common in consolidative‐type IMA (68.57%), of which G12V mutation was the most common, accounting for 40%; however, consistent with previous findings, none of the cases showed EGFR mutations.^18^, ^19^ Therefore, targeted drugs for KRAS, but not EGFR, mutations are eligible for patients with IMA.

In our study, 80.56% of the patients were initially diagnosed in the T3 and T4 stages, and only 16.67% of the patients were found with lymph node metastasis. This indicates that large consolidative IMAs do not tend to lymph node metastasis, which is consistent with the previous research results.^20^, ^21^ Because of the clinical insidious nature of the consolidative‐type IMA, the lesions were found to be large and the clinical stage was late initially, so prognosis was affected.

IMA has a better prognosis than non‐mucinous adenocarcinoma.^22^ Among IMA cases, the nodular type has a better prognosis than the pneumonic type.^23^ NIE et al.^24^ reported that the 5‐year RFS rates were 64.7% and 0% for the nodular and pneumonic‐type groups, respectively. Moreover, the pneumonic/consolidative type was an independent risk factor for prognosis.^25^ We found a poor prognosis of consolidative‐type IMA, with 3‐year RFS and OS rates of 30.0% and 75%, respectively. Watanabe et al.^18^ reported that the 5‐year OS and RFS rates of pneumonic‐type IMA were 20.0% and 0%, respectively. The inconsistency between our findings and those of Watanabe et al.^18^ could be attributed to differences in the definitions of consolidative‐type IMA, the pathological stages of the enrolled patients, and inevitable inclusion bias.

Because of the low incidence rate of IMA, and the lower incidence rate of large consolidative type on imaging, the sample size of this study is quite small. However, the sample size of this study is still higher than that of other studies in recent years, for example, Wang et al.^8^ recorded 26 cases of pneumonia‐type IMA; Huo et al.^10^ only recorded 21 cases of pneumonia‐type IMA; and Han et al.^17^ recorded 17 cases of pneumonia‐type lung cancer.

This study has several limitations. First, this was a single‐center retrospective study, which may involve inclusion bias and heterogeneous CT scan protocols. Second, we included a relatively small number of cases. Finally, some of the patients were lost to follow‐up. In conclusion, large consolidative‐type IMA tends to occur in the elderly population. It has diverse radiological manifestations, with frequent presentation of halo, angiographic, and dead branch sign. KRAS mutations are common, whereas EGFR mutations are rare. Patients initially diagnosed with large consolidative‐type IMA are usually in the T3–T4 stages; however, lymph node metastasis is rare. Finally, consolidative‐type IMA has a poor prognosis.

## AUTHOR CONTRIBUTIONS

Conceptualization: Jiaqi Chen, Linlin Qi, Jianwei Wang. Methodology: Jiaqi Chen, Linlin Qi, Jianwei Wang. Methodology validation: Jiaqi Chen, Linlin Qi, Jianwei Wang, Jianwei Wang. Investigation: Qi Xue, Jia Jia, Guochao Zhang. Data curation: Jiaqi Chen, Linlin Qi, Jia Jia, Guochao Zhang. Formal analysis: Jianing Liu, Fenglan Li, Shulei Cui. Writing—original draft: Jiaqi Chen, Linlin Qi. Writing—review and editing: Jiaqi Chen, Linlin Qi, Jianwei Wang. Visualization: Jiaqi Chen, Linlin Qi, Jianwei Wang, Jianwei Wang. Supervision: Jianwei Wang, Jianwei Wang, Qi Xue, Jia Jia, Guochao Zhang, Jianing Liu, Fenglan Li, Shulei Cui. Funding acquisition: Jianwei Wang. All authors contributed to the article and approved the submitted version.

## CONFLICT OF INTEREST STATEMENT

The authors declare that the research was conducted in the absence of any commercial or financial relationships that could be construed as a potential conflict of interest.

## ETHICS STATEMENT

This retrospective study was approved by the ethics committee of our institution, which waived the requirement for informed consent.

## Data Availability

Research data are not shared. The raw data supporting the conclusions of this article will be made available by the authors, without undue reservation.
